# The Rise in Mortality from Breast Cancer in Young Women: Trend Analysis in Brazil

**DOI:** 10.1371/journal.pone.0168950

**Published:** 2017-01-03

**Authors:** Sheila Cristina Rocha-Brischiliari, Rosana Rosseto de Oliveira, Luciano Andrade, Adriano Brischiliari, Angela Andreia França Gravena, Maria Dalva de Barros Carvalho, Sandra Marisa Pelloso

**Affiliations:** 1 Health Science Center, State University of Maringa, Maringa, Parana, Brazil; 2 Department of Nursing, State University of Maringa, Maringa, Parana, Brazil; 3 Department of Medicine, State University of Maringa, Maringa, Parana, Brazil; 4 Department of Post Graduate in Health Science, State University of Maringa, Maringa, Parana, Brazil; 5 Department of Post Graduate in Health Science and Nursing, State University of Maringa, Maringa, Parana, Brazil; University of South Alabama Mitchell Cancer Institute, UNITED STATES

## Abstract

**Introduction:**

Breast cancer is the most common cause of cancer death among women.

**Objective:**

The objective of this study was to analyze time trends in overall mortality from breast cancer in Brazil, Brazilian regions and States.

**Methods:**

This is an exploratory study, of the time series of deaths from breast cancer contained in the Mortality Information System (SIM), of women living in Brazil, Brazilian regions and States, from 1996 to 2013. For the trend analysis, the polynomial regression model was used, and a significant trend was considered when the estimated model obtained a p value <0.05.

**Results:**

There was a tendency of increased mortality from breast cancer in Brazilian women (average increase of 0.18 per year; *p* <0.001), with regional differences, particularly in the age group 20–49 years (0.07 per year; *p* <0.001). The age group 50–69 years remained constant but had high average rates (37.14).

**Conclusion:**

More effective planning is needed to focus on the different scenarios of the Brazilian regions. Screening strategies for the incidence and mortality from breast cancer must also be rethought according to age group in the country.

## Introduction

Breast cancer is a public health problem and is classified as the most common type of cancer among women. It is predicted that by 2020, breast cancer will be diagnosed in more than 1.97 million women worldwide, and 622.000 will die from this disease [1]. In Brazil, according to the National Cancer Institute (INCA), the number of new cases of breast cancer expected in 2016 is 57.960, accounting for 28.1% of all types of cancer in women [2]; and in 2013, the mortality was 14.206 women [3].

Although breast cancer still remains high in developed countries, there is a shift in the global distribution of cases, pointing out that breast cancer continues to emerge as a major health problem for women in Asia, Africa and South America [4] due to the growth and aging of the global population, as well as risk factors such as smoking, obesity and eating habits [5].

In Latin American countries, the mortality from cancer is in general, approximately twofold higher than it is in the more developed countries [6] and the incidence and mortality are likely to continuously increase in the coming decades [7], because of unprepared health systems to meet this grievance [8].

Brazil has a total area of approximately 8.5 million square kilometers, representing 47% of South America. With the current estimated population of 206 million [9], it is the fifth most populous country on the planet. Considering its size and the disparities found among the regions of the country, the death rates may vary between regions [10] depending on the distribution by age, sex, location and economic situation [7]. To date, the context of Brazilian disparity has been discussed infrequently, limiting further understanding of the impact of the disease [11,12], because knowing the cancer patterns in different populations is crucial to guide prevention efforts [8].

The mortality rate is one of the most important indicators for monitoring the health of patients with breast cancer [13], and trend analysis is a technique that aims to identify a pattern of changes or trends in several observations [14], both in countries and in specific regions, to inform local control strategies of the disease [5]. In this way, it aims to assume that the growth or decrease in cancer mortality has direct implications for the health assessment and may influence the review of prevention and control strategies [15] of cities, States or countries.

In this context, the aim of this study was to analyze the time trends in mortality from breast cancer in Brazil, regions and States. The analysis of timing trends contributes to gather epidemiological data that can be used to guide research and intervention programs of the early investigation of cancer determining factors. [5], reinforcing the potential of the health system for the early diagnosis and appropriate treatment of cancer [16], in the different regions of a country of continental dimensions such as Brazil.

## Methods

This is an exploratory study of the time series of deaths from breast cancer listed in the Mortality Information System (SIM), of women living in Brazil, Brazilian regions and States in the period 1996–2013.

We chose to perform the analysis using data 1996, the year in which the Mortality Information System started to record the causes of death according to the rules of the tenth revision of the International Classification of Diseases and Related Health Problems Statistics (ICD 10).

For the calculation of the mortality rates of breast cancer, the ratio was determined between the number of deaths from breast cancer in women and the female population in that year and location, obtained from the demographic information Census 2000, 2010 and estimates, multiplied by 100,000. Files with the mortality data were extracted from the Department of the Unified Health System (DATASUS) [17]. The female breast cancer mortality rates were further analyzed based on age groups: 20–49 years and 50–69 years. The choice to work with only women up to age 69 years was to prevent deaths due to other causes that could lead to false-positive results for mortality from this breast cancer.

For the trend analysis, the polynomial regression model was used in which the breast cancer rates were considered as dependent variables (y) and the years of study as an independent variable (x). The variable “year” was transformed into a year-centralized variable (x-2003), and the series were smoothed using a three-point moving average.

The polynomial regression models were tested as linear (y = β_0_+β_1_x_1_), quadratic (y = β_0_+β_1_x_1_+β_2_x_2_) and cubic (y = β_0_+β_1_x_1_+β_2_x_2_+β_3_x_3_), considering the significant trend that the estimated model obtained a *p value* < 0.05. To choose the best model, analysis of scatter-plots, the value of the coefficient of determination (r^2^) and residual analysis (real homoscedasticity assumption) were considered. When all of the criteria were significant for more than one model and the coefficient of determination was similar, the simplest model was chosen. Analyses were performed using SPSS software, version 20.1.

The cartographic basis of Brazil with States boundaries is publicly available online in shapefile (SHP) at the Brazilian Institute of Geography and Statistics (IBGE) website. Cloropleth maps were built to demonstrate the timeline distribution of the general mortality rates of breast cancer by age group among the Brazilian States. All of the figures were constructed using QGIS version 2.8 [18]. The spatial distribution of the breast cancer rates was presented in intervals, from the maximum to minimum rates, and the maps were depicted in green scales, setting the lighter colors for lower rates and darker colors for higher rates.

The research was approved by the Standing Committee on Ethics in Research of the State University of West Paraná—UNIOESTE (process number 1310870/2015). The data were obtained from public databases (http://datasus.saude.gov.br/).

## Results

**Fig 1:**
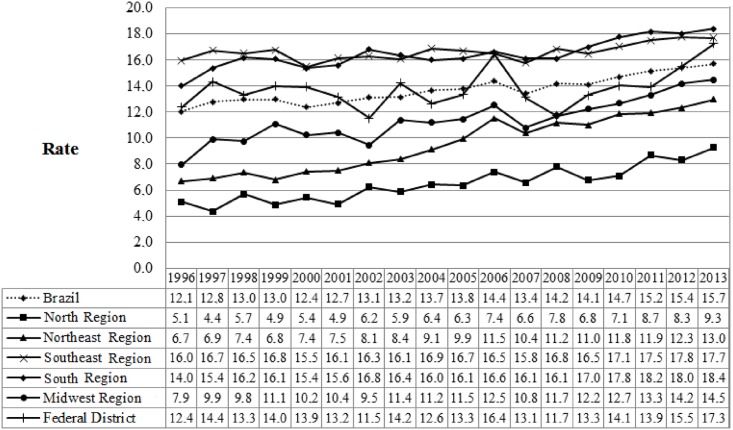
Mortality rate for breast cancer in women, according to place of residence, 1996–2013. (A) Presentation of mortality rate for breast cancer in Brazil and large regions along time period of eighteen years.

In total, 134,870 deaths from breast cancer were analysed from 1996 to 2013 in Brazilian women who were aged 20 to 69 years, white (57.25%), and married (49.96%), and who had an education less than eight years (38.3%). In 1996, the mortality rate from breast cancer in Brazil was 12.1, rising to 15.7 in 2013. Regarding the Brazilian regions, the Midwest and Northeast stood out increasing from 7.9 and 6.7 in 1996 to 14.5 and 13.0 in 2013, respectively (Fig 1).

**Fig 2:**
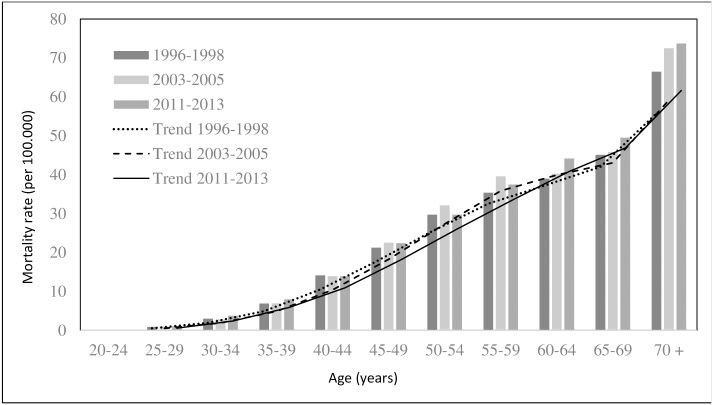
Breast cancer mortality in Brazil across age groups over three time periods. (A) The data of mortality from the evolution of breast cancer in Brazil were assembled by age group along three time periods.

Breast cancer mortality in Brazil over three time periods (1996–1998, 2003–2005 and 2011–2013) indicated in (Fig 2). The mortality remained high throughout the period.

The polynomial regression analysis showed an increasing trend of mortality from breast cancer in Brazil (increase of 0.18 each year; r^2^ = 0.92), as well as in the Brazilian regions where the highest average rates were found in the South (16.40) and Southeast (16.27). However the Northeast had the highest average increase (0.40 per year; r^2^ = 0.97) (Table 1).

**Table 1:** Trend models of mortality rates from breast cancer. Brazil, 1996–2013.

Local	Model	R^2^	p	Trend*
**Brazil**	**y = 13.60+0.18x**	**0.92**	**<0.001**	↑
**North Region**	**y = 6.34+0.23x**	**0.95**	**<0.001**	↑
Acre	y = 4.59+0.13x	0.12	0.185	-
Amazonas	y = 5.79+0.17x+0.05x^2^	0.94	<0.001	↓/↑
Roraima	y = 6.40+0.16x	0.16	0.128	-
Pará	y = 6.28+0.21x	0.87	<0.001	↑
Amapá	y = 5.23–0.34x-0.02x^2^+0.01x^3^	0.78	<0.001	↑/↓/↑
Rondônia	y = 6.98+0.32x	0.74	<0.001	↑
Tocantins	y = 6.01+0.34x	0.74	<0.001	↑
**Northeast Region**	**y = 9.28+0.40x**	**0.97**	**<0.001**	↑
Maranhão	y = 4.61+0.37x	0.93	<0.001	↑
Piauí	y = 7.70+0.64x	0.99	<0.001	↑
Ceará	y = 11.04+0.36x	0.91	<0.001	↑
Rio Grande do Norte	y = 9.42+0.40x	0.85	<0.001	↑
Paraíba	y = 8.32+0.56x	0.89	<0.001	↑
Pernambuco	y = 13.01+0.33x	0.83	<0.001	↑
Bahia	y = 8.47+0.38x	0.96	<0.001	↑
Alagoas	y = 7.85+0.47x	0.96	<0.001	↑
Sergipe	y = 10.25+0.57x	0.97	<0.001	↑
**Southeast Region**	**y = 16.27+0.06x+0.01x**^**3**^	**0.78**	**<0.001**	↓/↑
Minas Gerais	y = 11.55+0.23x	0.83	<0.001	↑
Espírito Santo	y = 12.38+0.43x	0.79	<0.001	↑
Rio de Janeiro	y = 21.35+0.27x+0.02x^2^-0.004x^3^	0.75	<0.001	↓/↑
São Paulo	y = 17.08–0.05x	0.25	0.047	↓/↑
**South Region**	**y = 16.40+16x**	**0.79**	**<0.001**	**↑**
Paraná	y = 14.37+0.24x	0.94	<0.001	↑
Santa Catarina	y = 13.96+0.22x	0.71	<0.001	↑
Rio Grande do Sul	y = 19.48+0.07x	0.40	0.009	↑
**Midwest Region**	**y = 11.24+0.25x**	**0.90**	**<0.001**	↑
Mato Grosso do Sul	y = 13.00+0.38x	0.70	<0.001	↑
Mato Grosso	y = 8.81+0.32x	0.75	<0.001	↑
Goiás	y = 10.59+0.26x	0.87	<0.001	↑
Federal District	y = 13.61+0.06x	0.16	0.121	-

* ↑ Increscent; ↓ Decrescent; - Constant; ↑/↓ Increscent/Decrescent; ↓/↑ Decrescent/Increscent; ↑/↓/↑Increscent/Decrescent/Increscent.

Regarding the States of North, Rondônia had the highest average (6.98), and Amapá had the highest annual average increase (0.34, r^2^ = 0.78); however, no significant trend was found in the States of Acre and Roraima (constant -; *p* = 0.185 and *p* = 0.128, respectively). In the Northeast, the State of Ceará had the highest average rate (11.4), and the State of Piauí presented the highest annual average increase among all Brazilian States (0.64, r^2^ = 0.99). In the Southeast, Rio de Janeiro stood out, with an average rate of 21.35 per 100,000 women and the State of Espírito Santo had the highest annual average increase (0.43, r^2^ = 0.79). It also showed that the State of São Paulo had a decrease in the year 2007, followed by an increase (-0.05 per year; r^2^ = 0.25). In the South region, the State of Rio Grande do Sul had the highest average rate for the period (19.48), and Paraná had the highest annual average increase (0.24, r^2^ = 0.94). In the Midwest, the Federal District had the highest average rate for the period (13.61), but no significant trend was found (constant; *p* = 0.121), and Mato Grosso do Sul had the highest annual average increase (0.38; r^2^ = 0.70) (Table 1).

Regarding age, in Brazil, there was a trend of increased mortality from breast cancer in women 20–49 years. The age group 50–69 years remained constant, but with high average rates. All of the regions showed an increasing trend in women 20–49 years, with the largest increase in the Northeast (0.19, r^2^ = 0.97) and higher average rates in the Southeast and South (7.64 and 7.40, respectively). Regarding the States, in the North, the highest average was in Rondônia (4.04), and the highest average annual increase was in the Tocantins (0.22, r^2^ = 0.60). In the Northeast, the highest rate was in Pernambuco (6.52), and the highest average annual increase was in Paraiba (0.88, r^2^ = 0.93). In the Southeast the highest rate was in Rio de Janeiro (10.23), which showed a steady trend (*p* = 0.230), and the highest average annual increase was in the Espírito Santo (0.17, r^2^ = 0.60). In the South the highest rate was in Rio Grande do Sul (8.34), and the highest average annual increase was in Paraná (0.06 r^2^ = 0.38) and Rio Grande do Sul (0.06, r^2^ = 0.42). In the Central West region, the State of Mato Grosso do Sul stood out, with the highest rate and average annual increase in the region (7.05; 0.11, r^2^ = 0.69) (Table 2).

**Table 2:** Trend models of the mortality rates from breast cancer according to age. Brazil, 1996–2013.

	20–49 years				50–69 years			
Local	Model	R^2^	p	Trend*	Model	R^2^	p	Trend*
**Brazil**	**y = 6.55+0.07x**	**0.91**	**<0.001**	**↑**	**y = 37.14x**	**0.25**	**0.055**	**-**
**North Region**	**y = 3.53+0.12x**	**0.90**	**<0.001**	↑	**y = 19.43+0.60x**	**0.92**	**<0.001**	↑
Acre	y = 2.58+0.08x	0.10	0238	-	y = 14.23+0.22x	0.05	0.396	-
Amazonas	y = 3.21+0.08x+0.03x^2^	0.88	<0.001	↑	y = 19.57+0.46x+0.15x^2^	0.79	<0.001	↓/↑
Roraima	y = 3.40+0.13x	0.23	0.059	-	y = 24.14–0.11x	0.004	0.816	-
Pará	y = 3.49+0.10x	0.80	<0.001	↑	y = 18.67+0.61x	0.73	<0.001	↑
Amapá	y = 2.89+0.12x	0.45	0.005	-	y = 16.55+0.38x	0.05	0.410	-
Rondônia	y = 4.04+0.18x	0.63	<0.001	↑	y = 23.62+0.77x-0.11x^2^	0.62	0.001	↑/↓
Tocantins	y = 3.32+0.22x	0.60	<0.001	↑	y = 17.23+0.72x	0.62	<0.001	↑
**Northeast Region**	**y = 4.87+0.19x**	**0.97**	**<0.001**	↑	**y = 24.42+1.02x**	**0.94**	**<0.001**	↑
Maranhão	y = 2.74+0.22x	0.92	<0.001	↑	y = 11.63+0.94x	0.91	<0.001	↑
Piauí	y = 4.29+0.30x	0.96	<0.001	↑	y = 19.04+1.66x	0.97	<0.001	↑
Ceará	y = 5.69+0.17x	0.83	<0.001	↑	y = 28.98+0.88x	0.87	<0.001	↑
Rio Grande do Norte	y = 4.43+0.16x	0.65	<0.001	↑	y = 24.77+2.25x0.10x^2^-0.03x^3^	0.96	<0.001	↑
Paraíba	y = 4.12+0.88x	0.93	<0.001	↑	y = 21.11+1.34x	0.81	<0.001	↑
Pernambuco	y = 6.52+0.08x	0.45	0.004	↑	y = 34.18+0.95x	0.79	<0.001	↑
Bahia	y = 4.59+0.20x	0.96	<0.001	↑	y = 22.05+0.86x	0.95	<0.001	↑
Alagoas	y = 4.39+0.26x	0.95	<0.001	↑	y = 20.58+1.13x	0.94	<0.001	↑
Sergipe	y = 5.76+0.31x	0.86	<0.001	↑	y = 27.17+1.36x	0.92	<0.001	↑
**Southeast Region**	**y = 7.64+0.01x+0.01x**^**2**^	**0.72**	**<0.001**	↓/↑	**y = 44.35–0.39x**	**0.63**	**<0.001**	↓
Minas Gerais	y = 5.30+0.11+0.01x^2^	0.96	<0.001	↓/↑	y = 30.63+0.21x	0.36	0.014	↑
Espírito Santo	y = 6.41+0.17x	0.60	0.001	↑	y = 33.46+0.81x	0.55	0.001	↑
Rio de Janeiro	y = 10.23+0.02x	0.10	0.230	-	y = 53.96–0.30x	0.49	0.003	↓
São Paulo	y = 7.88–0.04x	0.64	<0.001	↓	y = 47.24–0.77x	0.80	<0.001	↓
**South Region**	**y = 7.40+0.004x+0.01x**^**2**^	**0.66**	**0.001**	↓/↑	**y = 43.10–0.07x**	**0.04**	**0.439**	-
Paraná	y = 7.08+0.06x	0.38	0.011	↑	y = 39.90–0.06x	0.66	<0.001	↑/↓
Santa Catarina	y = 6.65+0.04x+0.02x^2^	0.76	<0.001	↓/↑	y = 37.27+0.13x	0.10	0.223	-
Rio Grande do Sul	y = 8.34–0.06x+0.01x^2^	0.42	0.011	↓/↑	y = 49.20–0.32x	0.45	0.005	-
**Midwest Region**	**y = 5.79+0.07x**	**0.62**	**<0.001**	↑	**y = 33.71+0.46x**	**0.60**	**0.001**	↑
Mato Grosso do Sul	y = 7.05+0.11x-0.03x^2^	0.69	<0.001	↑/↓	y = 41.34+1.08x-0.20x^2^	0.73	<0.001	↑/↓
Mato Grosso	y = 5.02+0.11x	0.25	<0.047	↑/↓/↑	y = 25.88+0.79x	0.92	<0.047	↑
Goiás	y = 5.45+10x	0.79	<0.001	↑	y = 30.79+0.43x	0.62	<0.001	↑
Federal District	y = 6.70–0.05x	0.19	0.087	-	y = 46.04–0.34x	0.13	0.169	-

* ↑ Increscent; ↓ Decrescent; - Constant; ↑/↓ Increscent/Decrescent; ↓/↑ Decrescent/Increscent; ↑/↓/↑Increscent/Decrescent/Increscent.

The breast cancer mortality trends in Brazil in the age group 50–69 years remained constant (*p* = 0.05). In Brazilian regions, the North, Northeast and Midwest showed a growing trend for women 50–69 years, with the highest increase in the Northeast (1.02, r^2^ = 0.94). In the Southeast, despite having the highest average rate for the period (34.35), there was a decreasing trend (-0.39 per year). In the South, the rates remained constant (*p* = 0.439).

Regarding the States in the North, the highest average (23.62) and highest average annual increase occurred in Rondônia (0.77, r^2^ = 0.62). In the Northeast, the highest average was in Pernambuco (34.18), and the highest annual average increase in Ceará (2.25, r^2^ = 0.87). In the Southeast, the highest average was in Rio de Janeiro (53.96) and the highest annual average increase was in the Espírito Santo (0.81, r^2^ = 0.55). However, in the States of São Paulo and Rio de Janeiro, there was a decrease in the death rates from breast cancer in 50 to 69 year old women (-0.77and -0.30, respectively). In the South, the highest average was in Rio Grande do Sul (49.20) but with a greater annual decrease (-0.32). In the Midwest, the highest average was in the Federal District (46.04), with no significant trend, and the highest annual average increase occurred in Mato Grosso do Sul (0.08, r^2^ = 0.73) (Table 2).

**Fig 3:**
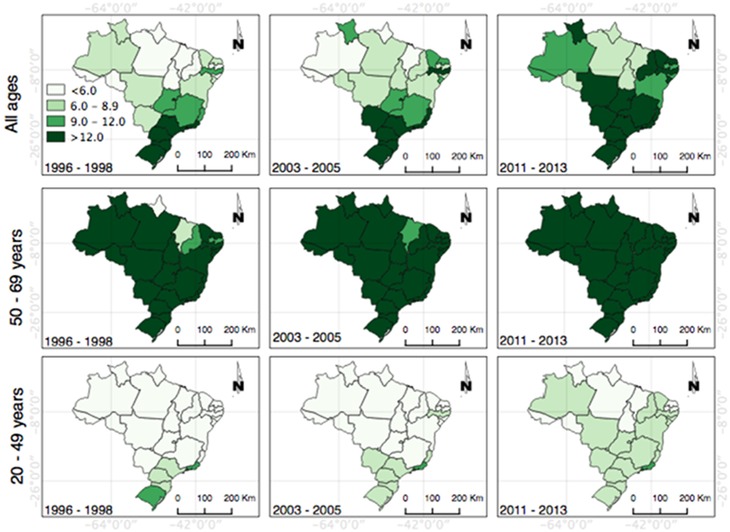
Distribution of the mortality rates from breast cancer in the Brazilian States. (A) Represents the thematic map of Brazil by regions and States and shows the distribution of the general mortality rates and age groups 20–49 years and 50–69 years in three-year periods (1996–1198, 2003–2005 and 2011–2013).

After visual inspection, it was observed that the highest rates were found in the last three years, indicating the increase in mortality from breast cancer in the country. It is clear that the regions and States that previously had low mortality from breast cancer in women had a significant increase (Fig 3).

## Discussion

Mortality distribution studies for breast neoplasias by time trends, based on regional disparities in Brazil, are still scarce despite being essential to the understanding of the disease. This study showed the increasing trend of mortality from breast cancer in women in Brazil, especially among young women, with variations in the five regions of the country and among the Brazilian States. Mortality from breast cancer among women 20–49 years showed a significant upward trend, while the rates of those 50 and 69 years remained constantbut high.

The incidence of cancer is increasing in developing countries [4]. In this study, of the five Brazilian regions, two stood out with a more significant annual average increase in mortality from breast cancer (Northeast and Midwest), corroborating data that have been previously described in the national literature from 1980 to 2009 [12]. For other regions, contradictory data were found in a Brazilian study with decreasing rates for the Southeast and stable rates for the South [19]. Another research has shown compliance with the present study of stabilization in the Southeast, with conflicting results for the South region, which showed a decrease [12].

The variation in the breast cancer mortality rates in the country may have a direct relationship with an early diagnosis and timely treatment, with significant differences in the clinical stage at diagnosis among patients seen by the public health system in relation to private health insurance [20], as well as behavioral and lifestyle influences [21], especially because of differences in risk factors [22, 23]. One hypothesis for regional variation found in the breast cancer mortality rates in Brazilian women may still be related to the lack of available resources for treatment in less developed States or the inaccessibility of most of the population to the treatment [11]. Brazil is a country of continental dimensions, with wide regional and social inequalities [24], and differences also noted in the health sector through different health indicators between Brazilian regions and States.

A recent study in the West Bank also showed regional variations in cancer mortality due to differences in social and cultural environments, including lifestyle and race, as well as variations in access to health services [25]. However, there are few publications that discuss issues of inequality in relation to the implementation of preventive practices based on regional characteristics [26].

A single Brazilian study that analyzed the trend of breast cancer to assess its association with social inequalities showed reductions in the rates in the more developed States, possibly reflecting better health care [11]. However, there are various factors that may contribute more to the increase or decrease in breast cancer than social inequality, especially in a country of large territorial extension.

A temporal trend study of mortality from breast cancer in the Northeast of Brazil found a strong upward trend with large developments in rates until 2030, making the structuring of promotion, surveillance and health care essential for this disease in this region [27]. As shown in this study, the Northeast and Midwest regions, which are economically disadvantaged, previously had a lower level of mortality from breast cancer in the country but had an abrupt increase and could negatively impact the number of deaths from this cause in the country in a few years.

By contrast, greater access to mammography and specialized treatment services seems to benefit women diagnosed at the early stages of the disease and may be associated with reduction in death rates from breast cancer in the Southeast and South [28], which could explain the stabilization in the Southeast, especially in the State of São Paulo, but not confirmed data in the present study, for the South.

Despite the efforts to reduce the disparities in cancer treatment, significant barriers persist in Latin American countries [6]. There is a need for a national dialogue on regional disparities and the implementation of equal access and use of a quality treatment for cancer, with early detection, genetic and genomics research, molecular subtyping of breast cancer, sensitizing specific actions and appropriate therapy [29]. It must be considered that developing countries such as Brazil are subjected to serious problems in access to health services, diagnosis and modern treatments [30], which contributes to the increased mortality rates from breast cancer between regions and States.

In Brazil, factors that deserve attention are the changes in reproductive factors in recent decades, with the increase in nulliparous women, a low fertility rate and the postponement of pregnancy to an older age [31]. This is due to the professional investment and seeking better living conditions, being an important association for the development of breast cancer [32].

Regarding age, a trend is shown in increased mortality in all regions of Brazil in women 20–49 years. The high mortality from breast cancer in the younger population is consistent with that in other studies that show an increasing trend in young women [33,34]. On the other hand, research in the Asia-Pacific region showed significant differences in mortality trends for breast cancer by age group, with a greater reduction in mortality rates for women younger than 50 years [4], a trend that may be related to screening where the early detection of cancer is possible before symptoms appear, providing better results [35].

The increase in the incidence of and mortality from breast cancer among young Brazilian women is a call to action [36]. In Brazil, the public health service gives priority to screening women aged over 50 years, and the occurrence of breast cancer in young women is still poorly understood [37]. There is a shortage of information in Latin America on the clinical and histological characteristics, gene expression, molecular patterns, prognosis, survival and risk factors among young women with breast cancer compared with what is known in the US and other developed countries [36].

In this sense, the trend is that the new cases of the disease diagnosed in advanced stages continue to emerge in younger women [38]. Thus, the clinical protocols and public policies that encourage this form of detection for young women are recommended in Brazil [37].

Despite showing a high mortality trend from 1996 to 2013 for women aged 50–69 years in all Brazilian regions and States, there is a need to consider that the polynomial regression analysis in the Northeast, Midwest and North showed greater tendencies to increase, with higher growth in the Northeast. These data may reflect factors already mentioned, such as the deficit in access to screening, leadsing a to late diagnosis of the disease, a worse prognosis and a consequent increase in mortality from breast cancer in these regions. Therefore, the data signal the inefficiency of public health policies regarding the conduct of strategies to control the incidence and mortality from breast cancer in the country.

The literature also notes that the older Latin American population after 2020 will be over 100 million people, a fact that will contribute to the increased incidence of cancer [6]. The study of breast cancer in Argentina highlights the major impact of demographic change on variations in mortality from this cancer [33].

According to a World Health Organization (WHO) report, the increase in income and improvements in living standards in developing countries have been accompanied by an increase in the incidence of breast cancer. This may be due to a longer life, greater exposure to risk factors, more fatty intake, obesity rise and lower pregnancy rates [39].

In this sense, personal, contextual and environmental factors need to be investigated in depth, and it is important that health records contain reliable and complete information so that they can become an adequate source of information as a tool for health policies and practices [25]. Identifying risk factors is important for future studies to elucidate the causes of any increasing or decreasing trend in each country [40].

One limitation of the study was the deficit of the information system in relation to factors associated with mortality from breast cancer that are restricted in the death record. However, performing mortality trend research for breast cancer in a country with large territorial proportions is essential to view the existing nuances between the variations of this causality.

It must also be considered that the collection of secondary data can be seen as a source of sensitive data, but the completeness of epidemiological variables in the Mortality Information System has shown improvement in deaths from breast cancer [41] and are fundamental for ecological studies in the country as the only source of available data on mortality. In this sense, the study of mortality data sets an important ally, as it allows us to describe the magnitude of the disease and serves as a tool to identify gaps in patient access to health services and indicate advances in treatment [42].

## Conclusion

Mortality from breast cancer showed a growing trend in Brazil, with increased emphasis on the Brazilian Northeast and Midwest, which until a decade ago had no significant relationship to this cause. However, Brazil's Northeast region had the largest increase among young women and among women of higher ages.

It was evidenced by the results of this study that mortality in younger women in the country (20–49 years) is gaining relevance and, in turn, the high rates in women ages 50–69 years persist. Thus, it is necessary to reconsider the recommendations of public health policies that establish the screening of breast cancer in relation to age, looking at the reality of each region. It is also necessary to consider the frequent changes that have occurred in people's habits and in socio-economic status and cultural development, as well as issues about access to diagnosis, treatment and strategic local planning, mainly for the early detection among younger women so mortality trends in breast cancer may decline in the country.

## Supporting Information

S1 Database According to the PLOS data policy, the authors provide the database as Support Information, containing the mortality rates from breast cancer in Brazil, by age and Brazilian region states. (XLSX)
